# An Early Myelosuppression in the Acute Mouse Sepsis Is Partly Outcome-Dependent

**DOI:** 10.3389/fimmu.2021.708670

**Published:** 2021-07-22

**Authors:** Tomasz Skirecki, Susanne Drechsler, Aldona Jeznach, Grażyna Hoser, Mohammad Jafarmadar, Jerzy Kawiak, Marcin F. Osuchowski

**Affiliations:** ^1^Laboratory of Flow Cytometry, Centre of Postgraduate Medical Education, Warsaw, Poland; ^2^Ludwig Boltzmann Institute for Experimental and Clinical Traumatology in the Allgemeine Unfallversicherungsanstalt (AUVA) Research Center, Vienna, Austria

**Keywords:** infection, cecal ligation and puncture, hematopoietic stem and progenitor cells, outcome prediction, immunity, caspases

## Abstract

Adult hematopoietic stem and progenitor cells (HSPCs) respond to bacterial infections by expansion to myeloid cells. Sepsis impairs this process by suppressing differentiation of stem cells subsequently contributing to an ineffective immune response. Whether the magnitude of HSPCs impairment in sepsis is severity-dependent remains unknown. This study investigated dynamics of the HSPC immune-inflammatory response in the bone marrow, splenic, and blood compartments in moribund and surviving septic mice. The 12-week-old outbred CD-1 female mice (n=65) were subjected to a cecal ligation and puncture (CLP) sepsis, treated with antibiotics and fluid resuscitation, and stratified into predicted-to-die (P-DIE) and predicted-to-survive (P-SUR) cohorts for analysis. CLP strongly reduced the common myeloid and multipotent progenitors, short- and long-term hematopoietic stem cell (HSC) counts in the bone marrow; lineage^−^ckit^+^Sca-1^+^ and short-term HSC suppression was greater in P-DIE *versus* P-SUR mice. A profound depletion of the common myeloid progenitors occurred in the blood (by 75%) and spleen (by 77%) of P-DIE. In P-SUR, most common circulating HSPCs subpopulations recovered to baseline by 72 h post-CLP. Analysis of activated caspase-1/-3/-7 revealed an increased apoptotic (by 30%) but not pyroptotic signaling in the bone marrow HSCs of P-DIE mice. The bone marrow from P-DIE mice revealed spikes of IL-6 (by 5-fold), CXCL1/KC (15-fold), CCL3/MIP-1α (1.7-fold), and CCL2/MCP-1 (2.8-fold) *versus* P-SUR and control (TNF, IFN-γ, IL-1β, -5, -10 remained unaltered). Summarizing, our findings demonstrate that an early sepsis-induced impairment of myelopoiesis is strongly outcome-dependent but varies among compartments. It is suggestive that the HSCPC loss is at least partly due to an increased apoptosis but not pyroptosis.

## Introduction

Adult mammals constantly produce mature blood cells from the hematopoietic stem cells in the process of hematopoiesis occurring in the bone marrow (BM) (1). In adulthood, the majority of hematopoietic stem and progenitor cells (HSPCs) are located in specialized niches in the BM, which protect them from oxidative injury and tightly control the hematopoiesis *via* multiple molecular interactions (2). However, even at the steady-state, a small subpopulation of HSCs circulates in the body *via* the blood and lymph in a process orchestrated by the sphingosine-1 phosphate gradient (3). It has been shown that through an expression of the toll-like receptors (TLRs), HSCs can sense microbial infections and locally differentiate to myeloid cells contributing to a local immune response (3, 4). An efficient fight with bacterial infections requires an increased production of short-lived myeloid cells that are constantly consumed from HSPCs in a coordinated process of the so-called emergency myelopoiesis (5). Microbial compounds are sensed by TLRs and nucleotide-binding oligomerization domain containing (NOD)-like receptors located on the endothelial cells of the BM niche, which in turn produce the granulocyte-colony stimulating factor (G-CSF) inducing proliferation and differentiation of HSPCs (6). In mice, G-CSF has been shown to drive mobilization of HSPCs from the BM niche to the blood and subsequently the spleen (7).

It is already well recognized from the murine models that sepsis profoundly affects the HSPCs compartment leading to an impaired myelopoiesis (8). Recognition of lipopolysaccharide (LPS) from gram-negative bacteria (*via* TLR4 receptor expressed by HSPCs) leads to an increased proliferation of HSCs with a concomitant blockage of differentiation into myeloid progenitor cells (9, 10). Importantly, the TLR4 signaling (*via* its adaptor TRIF) mediates a persistent injury to HSC self-renewal and repopulating functions—raising a question regarding the long-term effects of sepsis on hematopoiesis (11). We have reported a dysfunctional expansion of human HSCs following an abdominal sepsis in the humanized mice, demonstrating that this process was partially dependent on an increased Notch signaling (12). The dysfunctional myelopoiesis in the course of sepsis has been shown to especially affect neonatal and old animals (13, 14). Long-lasting epigenetic modifications of murine myeloid progenitor cells were recently shown to impair wound healing by their outgrowth macrophages underscoring negative effects of sepsis on the HSPCs compartment (15).

Currently, little is known about the hematopoiesis dynamics in septic patients, given that the blood serves as the sole source of information. In a previous study, we investigated the changes in circulating HSPCs in septic patients and observed an increased mobilization of primitive CD34^+^CD38^−^ and Lin^−^CD133^+^CD45^+^ HSCs early in the course of disease (16). The circulating HSPCs expressed markers of an active cell cycle, which appears to confirm the observations from septic mice. We also demonstrated that patients with a higher number of circulating HSCs are less likely to survive (16). Similarly, a negative correlation between circulating HSPCs and outcome was reported by Tsaganos et al. (17). However, neither the relationship between the BM HSPCs and sepsis severity/outcome nor the mechanistic link between dysfunctional hematopoiesis and sepsis course has been explored.

In this study, we hypothesized that the severity of sepsis is associated with the magnitude of changes in the early hematopoiesis. We analyzed the HSCP compartment in the BM, spleen, and blood harvested from outbred mice subjected to a clinically relevant model of polymicrobial sepsis by cecal ligation and puncture (CLP) and stratified into two homogenous predicted-to-die (P-DIE) and predicted-to-survive (P-SUR) cohorts. Our data provide a missing insight into the disturbances in emergency myelopoiesis that may lead to a potential therapeutic modulation of this process.

## Materials and Methods

### Mice

For the experiments 12-week-old female CD-1 (n=65) mice were purchased from Charles River Laboratories (Sulzfeld, Germany) and allowed to acclimatize to their new environment for at least 1 week prior to the experiment. Groups of five animals were housed in type III cages under standardized conditions (i.e., 12-h light-dark diurnal cycle, controlled temperature of 22–24°C). A standard rodent diet and fresh water were provided *ad libitum* throughout the study. Cages were enriched with carton houses, wooden boards, wood wool, hazel nuts, and wooden sticks for gnawing to facilitate natural behavior.

### Ethical Statement

All animal procedures were approved by the Viennese (Austria) legislative committee (Animal Use Proposal Permission No. 0899/2012/05) and conducted according to the National Institutes of Health guidelines.

### CLP Sepsis Model

All surgical procedures were executed under inhalation anesthesia with isoflurane (2-3%, Forane^®^, Baxter, Austria). Mice received analgesia (buprenorphine, bid, 0.05 mg/kg, s.c., Bupaq^®^, Richter Pharma, Austria), antibiotics (imipenem/cilastatin, Zienam^®^, bid, 25 mg/kg, s.c.), and fluid resuscitation (1 ml saline, 0,9%, bid) for the first five days after CLP.

Polymicrobial sepsis was induced using the cecal ligation and puncture model following the original protocol by Wichterman et al. (18) with modifications specified elsewhere (19). Shortly, anesthetized mice were placed in a supine position, abdomen was shaved, and skin disinfected. The abdominal cavity was opened, cecum exposed, ligated underneath the ileocecal valve with silk (Silkam^®^ 4.0, B.Braun), and punctured twice with an 18G needle. Abdominal wall was closed with single button sutures (Silkam^®^ 4.0), and skin was closed using skin adhesive (Histoacryl^®^, B.Braun). Antibiotic treatment was started 2 h after CLP surgery. Control mice did not undergo any surgery nor received treatment to serve as normal healthy mice reference.

### Study Design

**Figure 1:**
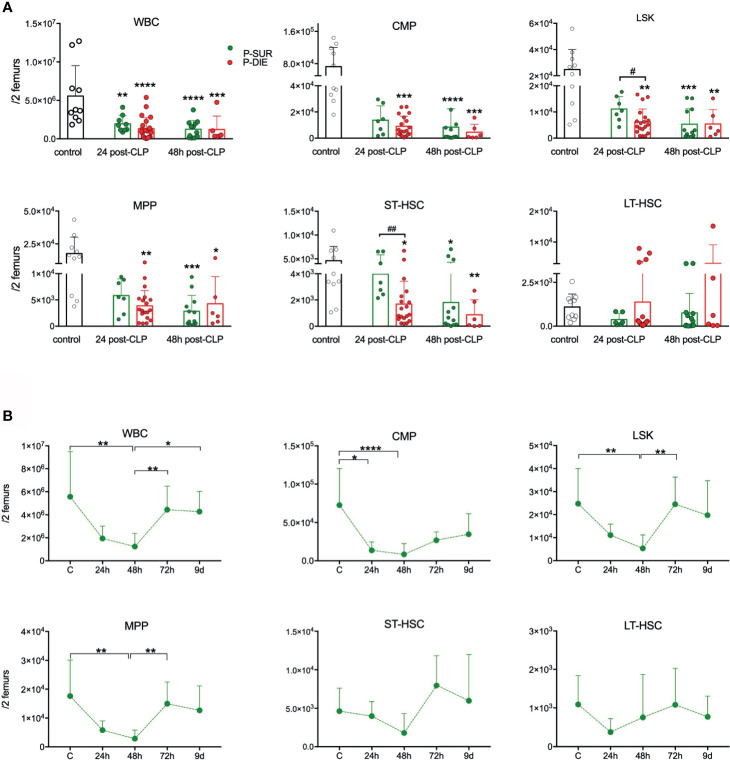
Outcome-related and longitudinal changes in the total cell count of the bone marrow progenitor cells in CD-1 mice subjected to CLP. **(A)** Comparative data for mice assigned as predicted to survive (P-SUR, green) and predicted to die (P-DIE, red) are shown for the white blood count (WBC), common myeloid progenitors (CMP), LSK cells, long-term hematopoietic stem cells (LT-HSC), short-term HSCs (ST-HSC), and multipotent progenitors (MPP). Subgroups were compared using Student t-test with Welch correction whenever required. Hash signs show differences between P-DIE and P-SUR groups; asterisks indicate differences between given subgroups of mice and control (healthy) mice, **(B)** Changes in the cell counts of given populations in the predicted to survive (P-SUR) mice only; dotted connecting lines indicate separate sets of P-SUR sacrificed at a given timepoint. Baseline (BL) n=10, 24 h n=9, 48 h n=12, 72 h n=9, 9 days n=5 Groups were compared using 1-way ANOVA with Tukey’s multiple comparison test. *p<0.05, **p<0.01, ***p<0.001, ****p<0.0001. ^#^p<0.05; ^##^p<0.01.

**Figure 2:**
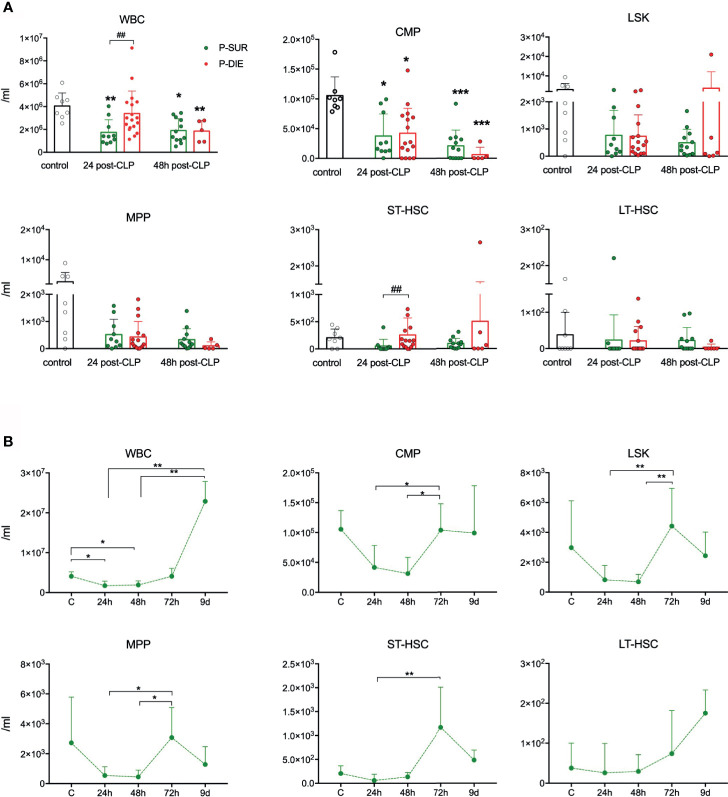
Outcome-related and longitudinal changes in the total cell count of peripheral blood progenitor cells in CD-1 mice subjected to CLP. **(A)** Comparative data for mice assigned as predicted to survive (P-SUR) and predicted to die (P-DIE) are shown for the white blood count (WBC), common myeloid progenitors (CMP), LSK cells, long-term hematopoietic stem cells (LT-HSC), short-term HSCs (ST-HSC), and multipotent progenitors (MPP). Subgroups were compared using Student t-test with Welch correction whenever required. Hash show differences between P-DIE and P-SUR groups; asterisks indicate differences between given subgroups of mice and control (healthy) mice, **(B)** Changes in the cell counts of given populations in P-SUR mice only; dotted connecting lines indicate separate sets of P-SUR sacrificed at a given timepoint. Baseline n=10, 24 h n=10, 48 h n=10, 72 h n=12, 9 days n=5. Groups were compared using 1-way ANOVA test with Tukey’s multiple comparison test. *p<0.05, **p<0.01, ***p<0.001. ^#^p<0.05.

**Figure 3:**
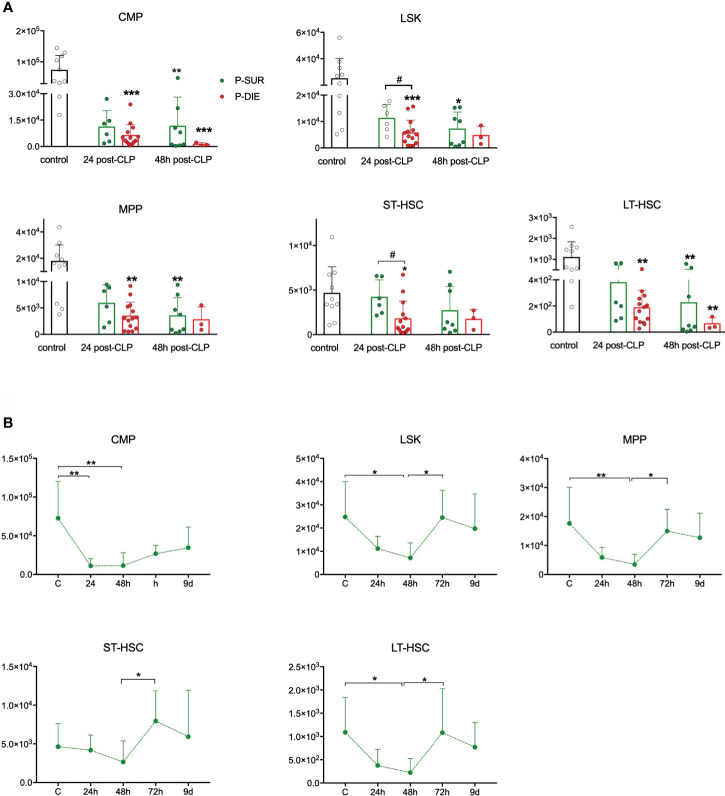
Outcome-related and longitudinal changes in the total cell count of the spleen progenitor cells in CD-1 mice subjected to CLP. **(A)** Comparative data for mice assigned as predicted to survive (P-SUR) and predicted to die (P-DIE) are shown for the common myeloid progenitors (CMP), LSK cells, long-term hematopoietic stem cells (LT-HSC), short-term HSCs (ST-HSC), and multipotent progenitors (MPP). Subgroups were compared using Student t-test with Welch correction whenever required. Hash show differences between P-DIE and P-SUR groups; asterisks indicate differences between given subgroups of mice and control (healthy) mice, **(B)** Changes in the cell counts of given populations in P-SUR animals only; dotted connecting lines indicate separate sets of P-SUR mice sacrificed at a given timepoint; baseline (BL) n=10, 24 h n=8, 48 h n=8, 72 h n=9, 9 days n=5. Groups were compared using one-way ANOVA test with Tukey’s multiple comparison test. *p<0.05, **p<0.01, ***p<0.001. ^#^p<0.05.

The study focused on differentiating lethal and surviving phenotypes for the hematopoiesis-related endpoints. To enable that, all CLP mice were stratified into two homogenous cohorts with high and low probability of survival: i.e., predicted-to-die (P-DIE) and predicted-to-survive (P-SUR). Mice identified as P-DIE at approximately 24 and 48 h (± 3 h of each timepoint) were euthanized and samples harvested. To ensure an appropriate outcome comparison, a limited number of P-SUR mice were simultaneously sacrificed at the same timepoints following the previously used protocol (19, 20). Mice that could not have been stratified into either P-DIE or P-SUR were monitored and sacrificed at later timepoints if they met either of those two criteria. We selected 24 and 48 h timepoints for P-DIE *vs.* P-SUR comparison based on the mortality dynamic of our CLP model: the bulk of CLP deaths occurred within the 48 h of CLP. Extending the P-DIE/P-SUR comparison to further timepoints would have radically increased the number of mice, and it would not have adhered to the 3R tenet. To most judiciously utilize the remaining CLP mice, we created longitudinal P-SUR-only profiles (shown in **Figures 1**–**3B**, **Supplementary Figures 1E–H**, and **Supplementary Figure 4**) by sacrificing P-SUR mice at 72 h and day 9 post-CLP.

The design of our study adheres to the majority of the points described in the Minimum Quality Threshold in Pre-Clinical Sepsis Studies (MQTiPSS) Consensus Recommendations (21); we met 17 points, failed to meet eight, whereas four were not applicable (**Supplementary Table 1**).

### Monitoring

All mice were monitored for clinical signs of illness and their status was evaluated using our custom-developed modified mouse clinical assessment scoring system (M-CASS) based on e.g. fur, posture, mobility, alertness, startle, righting reflex (and others) (22) starting 12 h post-CLP. Simultaneously, rectal temperature was monitored (Fluke Series II thermometer, Fluke, USA) at least twice daily (or more often whenever a mouse deteriorated) to ensure a maximally precise outcome stratification and humane endpoints. Mice were deemed moribund and assigned as P-DIE whenever the righting reflex was absent or/and M-CASS score ≥8 and/or body temperature (BT) < 28°C (recorded in at least two sequential measurements) and immediately euthanized under deep inhalation anesthesia with isoflurane followed by cervical dislocation. The BT-based prediction of outcome we have developed in our laboratory is highly accurate [AUC = 0.94 (19)] and was repeatedly used in previous studies (20, 22, 23).

### Blood Sampling and Blood Cell Count

A serial low-volume blood sampling was used to longitudinally monitor the changes in the peripheral blood cell counts (23) shown in **Supplementary Figure 1**. Briefly, 30 μl of blood was drawn by puncturing the facial vein with a 23-G needle, and blood was collected with a pipette. Samples were then immediately diluted 1:10 in PBS with ethylenediaminetetraacetic acid. After centrifugation (1,000*g*, 5 min, 22°C), 270 μl of plasma was removed and the remaining blood pellet was resuspended with 180 μl Cell-Dyn buffer with EDTA and a complete blood count with differential was performed with a Cell-Dyn 3700 counter (Abbott Laboratories, Illinois, USA).

### Immunophenotyping

Peripheral blood was obtained from mice under isoflurane anesthesia from retroorbital venous plexus. After cervical dislocation, the spleen and femurs were dissected. Red blood cells were lysed in all samples with ACK Lysing Buffer (ThermoFisher Scientific). Samples were stained with the following antibodies: anti-lineage cocktail-APC (clones: 145-2C11 (CD3e), M1/70 (CD11b), RA3-6B2 (CD45R/B220), TER-119 (Ly-76), RB6-8C5 (Ly6G/Ly-6C, BD Pharmingen, San Jose, CA, USA), anti-Sca-1 (Ly-6A/E)-PE-Cy7 (D7, BD Pharmingen), anti-ckit (CD117)-PE (2B8, BD Pharmingen), anti-CD48-biotin (clone HM48-1, BioLegend, San Diego, CA, USA), anti-CD150- AlexaFluor 488 (clone TC15-12F12.2, BioLegend). Following 20 min of incubation in room temperature, Streptavidin PE-eFluor610 (eBioscience) was added for next 15 min and cells were washed with 1 ml of PBS and resuspended in 0.5% formaldehyde in PBS. Cells were analyzed FC-500 flow cytometer (Beckman Coulter, Brea, CA, USA). A minimum of 50,000 mononuclear cells were recorded and analyzed in FlowJo software (FlowJo LLC., Ashland, OR, USA).

### Cytokine Assay

Both femurs from each mouse were dissected and then flushed with 0.5 ml of PBS each. Next, the bone marrow suspension was spun at 400*g* for 5 min, and supernatants were collected. Samples were stored at −86°C until analysis. The bone marrow concentration cytokines (IL-6, IL-8/KC, IL-10, TNF, MCP-1) was measured using Luminex Multiplex Immunoassay (Invitrogen, Thermo Fisher Scientific, Vienna, Austria) according to the manufacturer’s protocol. Finally, concentration of these mediators was normalized against the albumin concentration (Bradford Protein Assay, Thermofisher) in the BM samples to minimize the potential differences in harvesting BM from femurs.

### Apoptosis and Gene Expression Analysis

The bone marrow cells were obtained and subjected to erythrocyte lysis as described above. Next, samples were split for the caspase-1 and caspase-3/7 analysis. For the evaluation of activated caspase-1, BM cells were incubated for 30 min with the FAM-YVAD-FMK FLICA Caspase-1 reagent (ImmunoChemistry Technologies, LLC, MN, USA) according to the manufacturer’s protocol and then stained with the anti-Lineage -APC, anti-ckit-PE, and anti-Sca-1-PE-Cy7 antibodies as described. Activated caspases-3/7 were analyzed by staining with the FMK-DEVD FLICA probe (Vybrant FAM caspase-3 and-7 Assay, Thermofisher) and then co-stained with surface antibodies as described above.

For the gene expression flow cytometry analysis, lysed bone marrow cells were stained with the eBioscience Fixable Vability Dye eFluor 506 (Thermofisher) and the following antibodies: anti-lineage eFluor 405 (Thermofisher), anti- Sca-1 (Ly-6A/E)-PE-Cy7, and anti-ckit (CD117)-PE (both BD Pharmingen). Then cells were fixed, permeabilized, and hybridized with probes against TNF, IL6, and IL1β mRNA using the PrimeFlow RNA Assay Kit (Thermofisher) according to the manufacturer’s protocol. As a positive controls additional samples were stained against β2-microglobulin. Cells were analyzed with FACSCanto II flow cytometer (BD), and FlowJo software was further used to analyze the results.

### Statistical Analysis

Normality of all data sets was assessed using the Shapiro-Wilk test and log-normally transformed whenever necessary to achieve Gaussian distribution and control for existing outliers. Comparisons between P-DIE and P-SUR group were performed by t-test (with Welch correction for unequal variances if needed) and Mann-Whitney (for non-Gaussian data distribution) at each timepoint separately given that the P-DIE group did not meet assumptions (non-random deaths) for a repeated measures testing. The comparison of variables in P-SUR mice at different timepoints was performed using the two-way ANOVA with Tukey’s post-hoc test. p<0.05 was considered significant. Data are shown on the original scale as scatter plot overlaid over bars and expressed as means and standard deviations (SD), if not otherwise stated. GraphPad Prism 7 (GraphPad, Inc., USA) software was used for evaluating the statistical significance and/or graphical depiction of the data.

## Results

### A Transient Association of the Peripheral Blood Cell Counts With CLP Outcome

Based on the utilized outcome prediction, CLP resulted in approximately 40% mortality. Using the low-volume blood sampling, we monitored changes in the peripheral blood cell counts without sacrificing mice. Sepsis induced an early generalized peripheral leukopenia (mostly because of the depletion of neutrophils and lymphocytes) and lasted for at least 72 h post-CLP (**Figure S1**). Outcome-related differences were noted at 6 h (platelets lower in P-DIE; **Figure S1D**) and 24 h (lymphocytes higher in P-DIE *vs.* P-SUR; **Figure S1C**) post-CLP. Longitudinal analysis of the blood cell counts in the surviving mice showed a maximal decrease of circulating cells of all lineages at 24 h followed by a slow rebound until day 9 post-CLP (**Figures S1E–H**).

### CLP Outcome Is Associated With Depletion of the Hematopoietic Stem and Progenitor Cells (HSPCs) in the Bone Marrow

We utilized flow cytometry staining with a set of validated immunophenotype markers that were previously shown to be stably expressed in inflammatory conditions (7, 10). We used a standard gating strategy to distinguish major populations of murine HSPCs, including common myeloid progenitors (CMP; Lineage^−^ckit^+^Sca-1^−^), LSK (Lineage^−^ckit^+^Sca-1^+^), long-term repopulating stem cells (LT-HSCs; Lin^−^ckit^+^Sca-1^+^CD150^+^CD48^−^), short-term repopulating stem cells (ST-HSCs; Lin^−^ckit^+^Sca-1^+^CD150^+^CD48^+^), and multipotent progenitors (MPPs; Lin^−^ckit^+^Sca-1^+^CD150^−^CD48^−^) (**Figure S2A**) (10, 24, 25).

Although the cellularity of BM in control mice was spread (5.5 × 10^6^ +/−3.9 × 10^6^), CLP mice had a decreased total BM cell count in both P-SUR (1.9 × 10^6^) and P-DIE (1.3 × 10^6^) groups at 24 h; CMP suppression was evident in all groups at both timepoints (**Figure 1A**). The LSK, MPP, and ST-HSC counts were initially reduced in an outcome-dependent fashion: at 24 h, the suppression was more pronounced in P-DIE (*vs.* P-SUR) mice followed by a similar decrease at 48 h post-CLP (**Figure 1A**). In addition, we analyzed the CLP-induced changes in the frequency of given HSPCs subpopulations. Sepsis caused a significant decrease of the myeloid progenitor cell’s frequency only in P-SUR (but not P-DIE) mice at 24 h post-CLP (**Figure S2B**). The percentage of LSK and more specified MPP progenitors increased in P-DIE mice, whereas the subpopulations of ST-and LT-HSCs remained unaltered (**Figure S2B**).

We simultaneously monitored hematopoiesis changes in the BM of P-SUR mice until day 9. The strongest reduction in each analyzed cell population (except LT-HSCs) occurred at 48 h and was followed by a rapid recovery (to control values), indicative of their robust proliferation within the 48- to 72-h post-CLP interval (**Figure 1B**). Noteworthy, even at day 9 of observation, the myeloid progenitors count was, on average, two-fold lower compared with healthy mice.

### A Limited Effect of CLP Upon the HSPCs Counts in the Blood

As mobilization of HSPCs to circulation from the BM was shown in several infectious conditions in mice (4, 7, 16), we also studied stem cells in the peripheral blood after CLP (**Figure S3A**). No HSPC subpopulations (except CMP progenitors) were markedly diminished in the blood of septic mice (**Figure 2A**). Regarding outcome-based differences, only the subpopulation of ST-HSCs was 5-fold higher in P-DIE *vs* P-SUR mice 48 h after CLP, albeit not different from control (**Figure 2A**). In surviving mice (similarly to the kinetics observed in the BM), the subpopulations of HSPCs in the circulation were also prone to a robust rebound after 48 h despite the lack of an early post-CLP decrease (**Figure 2B**). Although leukopenia is not a common feature in septic patients, these results adhere to our previous observations showing lack of HSCs mobilization on the first day of ICU admission of septic shock patients (16).

### A Profound CLP-Induced Depletion of Splenic HSPCs Is Partly Outcome-Dependent

It was shown in a nonlethal *E. coli* infection that murine LSK cells migrate from the BM and accumulate in the spleen (7). We analyzed the splenic HSPCs to verify whether a similar process occurs in CLP (**Figure S3B**). We observed a profound depletion of CMPs at both post-CLP timepoints, irrespective of the outcome (**Figure 3A**). In LSK and ST-HSC progenitors, the early depletion was markedly outcome-dependent, LSK were 50% and ST-HSCs 60% lower in P-DIE *versus* P-SUR mice at 24 h (**Figure 3A**). In contrast to BM (**Figure 1A**), the rare LT-HSCs subpopulation was depleted in P-DIE mice at both post-CLP timepoints and in P-SUR mice at 48 h (**Figure 3A**).

The kinetic of the splenic HSPCs counts in P-SUR was similar to the one in the BM; both regarding their initial abrupt decrease (nadir at 48 h), as well as a robust numerical recovery of all (except CMP) cell subpopulations (**Figure 3B**). Although on day 9 after CLP, the spleens were enlarged (data not shown), the number of Lin^−^ckit^+^Sca-1^−^ CMPs did not reach the baseline level. Together, these data show that in an early sepsis, splenic HSPCs (including LT-HSCs) are severely depleted in an outcome-dependent manner.

### P-DIE Mice Show a Strong Pro-Apoptotic Signaling in the Bone Marrow HSPCs

**Figure 4:**
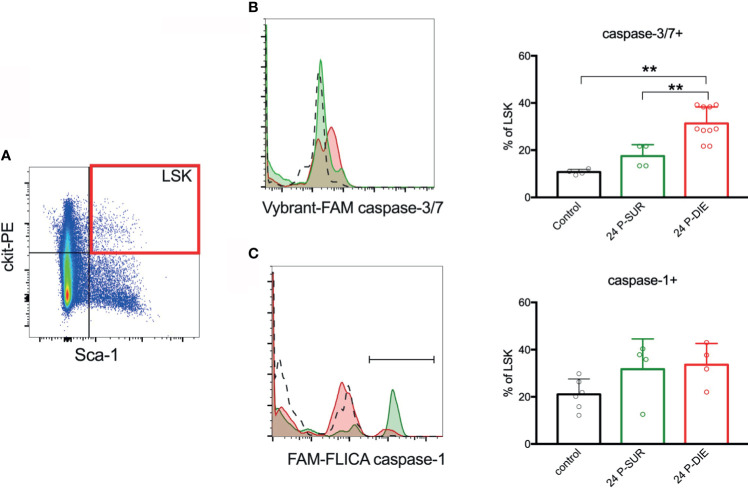
Activation of inflammatory and apoptotic caspases in the bone marrow hematopoietic stem and progenitor cells. **(A)** Lineage-negative mononuclear cells were analyzed for the expression of ckit and Sca-1 and the LSK subpopulation was analyzed for: **(B)** active caspases-3/-7 and **(C)** active caspase-1. Percentage of LSK cells with active caspases were compared between predicted to die (P-DIE) and predicted to survive (P-SUR) mice using 1-way ANOVA test with Tukey’s multiple comparison test. **p<0.01.

To gain insight into the mechanisms responsible for the acute depletion of LSK cells in septic mice, we analyzed hallmarks of apoptosis and pyroptosis at 24 h post-CLP (the timepoint preceding the nadir HSPC depletion). Caspase-3 and -7 activity in LSK cells from P-DIE mice was approximately 30% higher than that in P-SUR animals and three-fold higher compared with control (**Figures 4A, B**). Antibody-based analysis suggests increase in the cleavage of caspase-3 in LSK cells from P-DIE mice in comparison to P-SUR counterparts (**Figure S4**). We have utilized the FAM-FLICA probe to detect active caspase-1, which is a hallmark of inflammasome activation leading to pyroptosis. No meaningful inter-group differences for caspase-1 were detected (**Figures 4A, C**). An increased apoptosis appears to be the major mechanism of the HSPCs loss after CLP.

### Outcome-Related Changes in the Bone Marrow Cytokine Milieu After CLP

**Figure 5:**
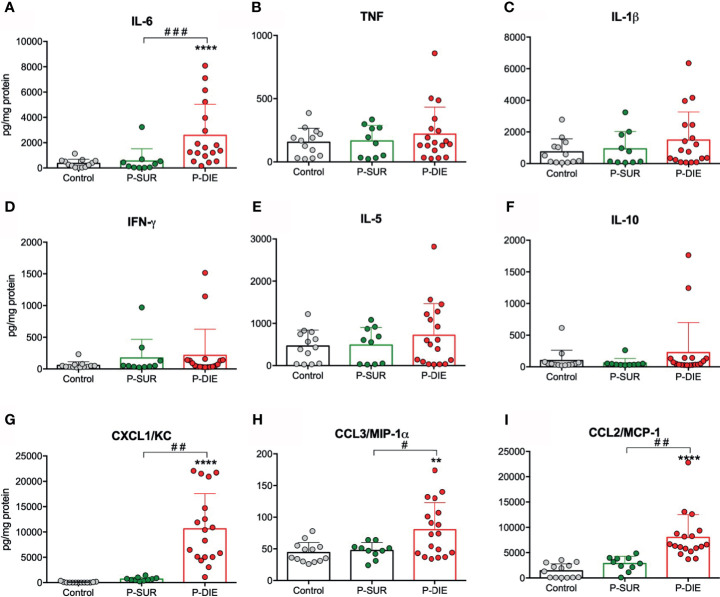
Outcome-related changes in the cytokine’s milieu of the bone marrow twenty-four hours after CLP. Total-protein normalized concentrations of **(A)** IL-6. **(B)** TNF. **(C)** IL-1β. **(D)** IFN-γ. **(E)** IL-5. **(F)** IL-10. **(G)** CXCL1/KC. **(H)** CCL3/MIP-1α. **(I)** CCL2/MCP-1 in the bone marrow supernatants of predicted-to survive (P-SUR) and predicted-to die (P-DIE) mice are shown. Concentration of cytokines between groups were compared using Student t-test with Welch correction whenever required. **p < 0.01, ****p < 0.0001 between control and septic mice. ^#^p < 0.05, ^##^p < 0.01, ^###^p < 0.001 between P-DIE and P-SUR groups.

We have previously shown that the systemic cytokine and chemokine response is strongly outcome-dependent in both early and late sepsis (26). Therefore, we analyzed selected cytokines in the BM supernatant from P-DIE and P-SUR mice 24 h after CLP. Similar to the circulating cytokine dynamics (26), IL-6 measured in the BM of P-DIE mice was approximately five-fold higher compared with P-SUR (2578 *vs* 550 *vs* 364 pg/ml in control mice; **Figure 5A**). In contrast, concentrations of IL-1β, IFN-γ, TNF, IL-5, and IL-10 remained unchanged in all three groups (**Figures 5B–F**). The outcome-dependent chemokine response was very robust: CXCL1/KC was 15-fold, CCL3/MIP-1α 1.7-fold, and CCL2/MCP-1 2.8-fold higher in P-DIE compared with P-SUR mice (**Figures 5G–I**).

We also analyzed the same cytokines/chemokines in P-SUR mice up to day 9 post-CLP. Only CXCL1/KC peaked at 24 h returning to the baseline level 24 h later (**Figure S5G**). Notably, we observed a uniform increase in all other mediators by day 9 after CLP; this change reached significance in IFN-γ (approximately 11-fold) and IL-10 (six-fold *vs* baseline; **Figures S5D, F**).

**Figure 6:**
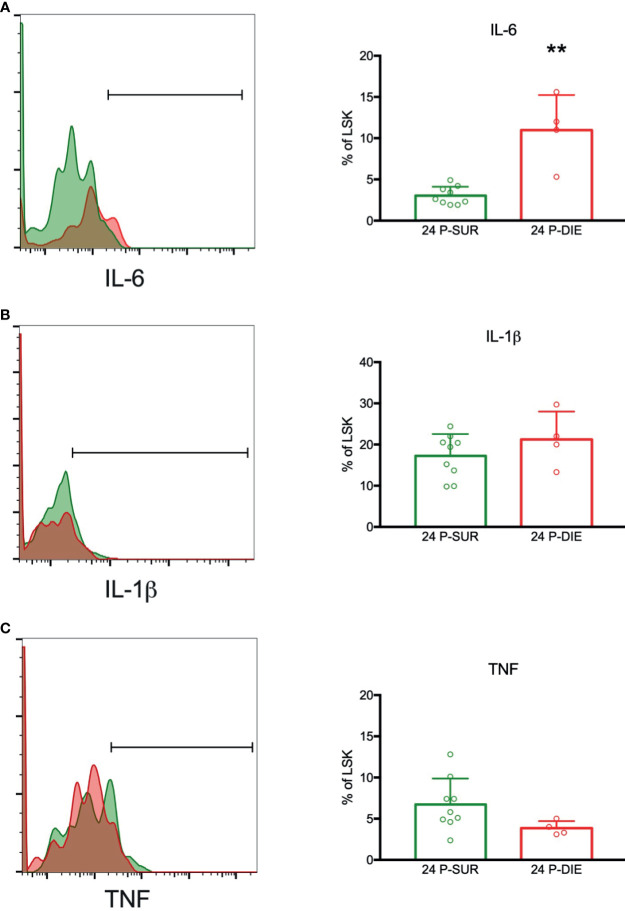
Expression of mRNA for selected cytokines by the bone marrow hematopoietic progenitors in sepsis. The LSK cells were analyzed twenty-four hours after CLP for the expression of mRNA transcripts for: **(A)** IL-6. **(B)** IL-1β. **(C)** TNF by flow cytometry. Groups were compared using Student t-test. **p<0.01.

Hematopoietic stem cells have been shown to produce significant amounts of cytokines in response to the toll-like receptor-mediated stimulation (27). We sought to establish whether HSPCs contribute to an increased production of cytokines in dying septic mice. To analyze the gene expression of IL6, IL1β, and TNF in the infrequent LSK cells, we utilized a flow cytometry-based technique to stain mRNA transcripts with gene-specific probes and branched DNA amplification (28). The LSK cells expressed mRNA for IL6, IL1β, and TNF (**Figure 6**). In cells from P-DIE mice, IL-6 transcripts were upregulated by three-fold in comparison with P-SUR, whereas no differences were present for IL-1β and TNF expression (**Figure 6**). These results indicate that the BM cytokine milieu is selectively altered in dying septic mice and HSPCs also contribute to this differential localized cytokine production.

## Discussion

This mouse study addressed a gap in human sepsis by investigating whether the HSPCs in their bone marrow niche are differentially affected with regard to outcome (i.e., dying *versus* surviving). Such a design approach is superior to the most commonly used healthy-*versus*-septic comparison given that it characterizes homogenous phenotypes specific for recovery and death (rather than an unspecific and heterogeneous disease signal). Using a clinically relevant model of polymicrobial sepsis, we demonstrated that the subpopulations of hematopoietic progenitors are depleted to a higher degree in mice predicted to die. We also identified apoptosis as the primary death pathway contributing to this effect. Simultaneously, the cytokine milieu of the bone marrow was more severely affected in the P-DIE animals and hematopoietic progenitors participated in synthesis of the IL-6.

Dysfunction of the bone marrow hematopoiesis in sepsis has been already shown by us and others and was mainly reported to be related to the block of differentiation of HSCs into myeloid progenitors (9, 12, 29). However, these changes were exclusively studied in pooled septic animals regardless of their clinical severity. In this study, we analyzed changes in several subpopulations of HSPCs in relation to sepsis severity in individual mice. Our results confirmed a depletion of percentage of myeloid progenitor cells in septic mice, which was more profound in mice with favorable prognosis. The frequency of bulk population of LSK cells (containing all hematopoietic stem cells) was expanded in P-DIE mice only. Also, subpopulations of multipotent progenitors and long-term repopulating HSCs expanded only in P-DIE mice. However, a total cell count evaluation of the HSPC subpopulations revealed contrasting dynamics, e.g., P-DIE mice had a markedly reduced number of myeloid progenitors, LSK cells, MPPs, and ST-HSCs. These results oppose some previous works reporting an expansion of LSK cells in sepsis (9, 10). However, others observed an increase in the frequency of LSK cell but without reporting the absolute counts (13, 29). There are several possible reasons for such discrepancy. First, we used outbred CD-1 mice that better recapitulate the background genetic heterogeneity present in patients and in turn an individual heterogeneity in response to a polymicrobial infection. Most of the above-cited studies used C57BL/6 (9, 10) and BALB/c (29) strains, which were different for various endpoints in response to sepsis compared with outbred mice. Second, mortality of those sepsis models was not defined, and sepsis severity (e.g. mild *vs* lethal) greatly modulates immune-inflammatory responses (30). Importantly, Kobayashi et al. (31), who also showed a CLP-induced LSK expansion, reported no decrease of BM cellularity, which is in striking contrast to severe leukopenia recorded in our study. Sepsis and LPS were shown to induce HSCs impairment by decreasing their self-renewal, repopulating, and myeloid differentiation ability by a direct TLR4 signaling (10, 11). TLR-mediated signaling was shown to induce HSCs proliferation (10, 11); although we did not perform a cell-cycle analysis, it cannot be excluded that our model triggered a similar response despite a numerical HSC loss. In P-SUR mice, the numerical cell recovery was typically present at 72 h post-CLP, suggestive of an enhanced proliferation induction, and the HSPC expansion was evidently halted by day 9. We conclude that physiological mechanisms controlling the HSPC dormancy-renewal balance (24) function properly even in a semi-lethal sepsis phenotype. Importantly, emerging evidence suggests that sepsis induces epigenetic modifications in HSPCs (15), but their exact role remains to be elucidated. Our study expands those earlier findings by exploring an individualized outcome-dependent loss of HSPCs. Yet, the causal relationship between HSCs impairment and its contribution to an early mortality in sepsis remains uncertain. It can be anticipated that in the early phase of sepsis, depletion of HSPCs is one of the many effects of maladaptive inflammatory response but this relationship can be more casual in the chronic phase as suggested by beneficial effects of CD34^+^ HSPCs transfer after CLP (32).

Depletion of HSPCs in BM can be attributed to either their mobilization to circulation or death. Notwithstanding, analysis of the peripheral blood revealed a decreased number of circulating HSPCs. Interestingly, the P-DIE mice had a five-fold higher number of circulating ST-HSCs in comparison to P-SUR animals. This reproduces our results from septic patients in whom a higher number of Lin^−^CD133^+^CD45^+^ HSCs was associated with diminished survival (16). In systemic infections, the spleen can become a site of extramedullary hematopoiesis (33). CLP induced a loss of all the HSPCs subpopulations, and the depletion was more profound in P-DIE mice. Even several days after CLP (with a pronounced splenomegaly present), the total number of any HSPCs subpopulations was not elevated. Although we did not perform analysis of MDSCs, it is conceivable that splenic HSPCs contribute to a local generation of these cells (34, 35). Remick and colleagues showed increased neutrophil and macrophage counts in the peritoneum of P-DIE mice after sepsis (36, 37). It can be speculated that some HSPCs also infiltrate the infection site, contributing locally to the production of those effector cells and cytokines. Particularly, despite failure to control bacterial infection, the P-DIE mice were shown to produce high levels of CXCL1 and CXCL2 in the peritoneum and blood that promoted neutrophil infiltration (36). As both chemokines are also chemoattractant for HSPCs (38), these cells are likely to follow a similar route. Burberry et al. (7) showed that systemic *E. coli* mobilizes HSCs to the spleen from BM in G-CSF-dependent manner, and this effect lasted several days after infection. However, their model was nonlethal and induced by a single pathogen — greatly different from a surgically induced polymicrobial sepsis that we used. It can be hypothesized that in P-DIE mice, the enhanced egress of HSPCs from their professional BM niches to other sites with higher oxygen tension and inflammatory stimulation drives functional impairment of their outgrowth cells [e.g., bactericidal capacities (37)]. As reported by Davis et al. (15), sepsis induces epigenetic modifications of HSPCs that attenuate monocyte functions in a long-term period (15). Evidence supporting this hypothesis were generated by Brudecki et al. (32) who showed that transfer of HSCs from healthy animals improves long-term survival of septic mice (32). However, early HSCs were reported to have also immunoregulatory function *via* TNF secretion (39). Whether such a mechanism contributes to immunosuppression in sepsis remains to be verified. In contrast, in mild, non-lethal infections migratory HSPCs contribute to bacterial clearance (40). Our observations complement the emerging picture of a global depletion of hematopoietic precursors in early days of septic shock.

Our findings also show activation of caspase-3 and -7 in BM HSPCs in P-DIE mice only indicating an activation of HSPC apoptosis in a severity-dependent manner — this is consistent with reports on detrimental role of apoptosis in sepsis (41, 42). Induction of apoptosis in HSPCs subpopulations after CLP was also reported by Zhang et al. (10). Intriguingly, caspase-3 was reported to also play non-apoptotic role in HSCs, e.g., to control their dormancy by limiting ERK signaling in response to some cytokines (43). It can be hypothesized that caspase-3 increase can also contribute to the low HSPCs counts by inhibiting their responsiveness to cytokine signals, but we only assessed the cleaved caspase-3 at 24 h. As HSCs were shown to be able to undergo pyroptosis *via* NLRP1 inflammasome (44), we measured activation of caspase-1 in LSK cells. Although we observed a relatively high activity of caspase-1 in sham mice, sepsis did not increase it further, and there was no significant increase in IL-1β in the BM. It is a rather unexpected finding given that neonatal mice with caspase-1/-11 knock-out showed an increased frequency of LSK cells in septic mice compared to WT littermates (45). The effects of HSPCs-targeted inhibition of caspase-1 and -3 in sepsis await characterization.

To the best of our knowledge, this study characterizes for the first time the changes in the BM cytokine milieu in early sepsis. Importantly, a robust increase in IL-6, CXCL1, MIP-1α (CCL3), and MCP-1 (CCL2) occurred in P-DIE mice only. In contrast to a previously reported steep rise in the blood (26), there was no elevation of TNF, IFN-γ, IL-5, IL-10 in the BM of P-DIE mice at 24 h post-CLP. IL-6 is a known factor inducing proliferation of HSCs (46); recently, its receptor has been shown to be upregulated *via* Notch-dependent manner upon TLR stimulation of HSCs (47). Aside from its proinflammatory profile, MIP-1α inhibits proliferation of HSCs and governs myeloid differentiation (48, 49). However, MIP-1α has been shown to inhibit formation of granulocyte-macrophage colony forming cells from human BM cells and can, therefore, appear as another factor contributing to a repression of HSPCs in sepsis. Secretion of MCP-1 (CCL2) by BM stromal cells is part of physiological response to infection supporting mobilization of inflammatory monocytes (50) but its local effect on HSPCs in sepsis remains unknown. The net effects of these mediators on HSPCs in sepsis require further studies. Interestingly, in P-SUR mice 9 days after CLP, there was a marked increase in INF-γ and IL-10 in the BM milieu. During a viral infection, INF-γ derived from CD8^+^ cytotoxic T cells was shown to promote myeloid differentiation by stimulation of IL-6 secretion from the BM niche cells (51), but the CLP mice did not show upregulation of IL-6 at the time of INF-γ peak. Previously, we have shown that BM preserves INF-γ-producing CD4^+^ memory T cells (52), and it can be speculated that both CD8^+^ and CD4^+^ T cells populations contribute to the INF-γ release but its role remains unknown. Using the flow cytometry-based mRNA assay, we confirmed the ability of LSK cells to express IL-6, TNF, and IL-1β (27). In line with the bulk protein analysis, IL-6 transcript was upregulated in P-DIE mice. Although neither the frequency of cytokine-producing LSK cells nor their numbers are high, these cells were shown to be potent IL-6 producers, thereby participating in HSPCs proliferation and differentiation (27). Our results further indicate occurrence of this mechanism during sepsis.

Our study is not limitation-free. We performed the experiments only in female mice and failed to compare our findings in septic males. Moreover, we did not perform functional HSCs assay but relied on the combination of previously validated immunophenotype markers (7, 10, 29). Analysis of HSPCs at the infection site, e.g. peritoneum would be of interest, but we were unable to establish a reliable analysis of LSK cells in probes from the abdominal cavity. Although our model was characterized by the mortality similar to the one reported in septic shock patients (53), most CLP deaths occurred within forty-eight hours (despite fluid resuscitation and antimicrobial treatment). This limited our outcome-related comparative analysis to early sepsis; it is imperative to analyze hematopoiesis in chronic sepsis given that any long-term hematopoietic deficits may contribute to the common post-sepsis sequelae.

Summarizing, we demonstrated that the hematopoietic stem and progenitor cells compartments are more profoundly diminished in P-DIE mice albeit this difference is not all-inclusive but cell population-selective. Depletion of hematopoietic precursors in early CLP sepsis constitutes a systemic feature as we did not observe mobilization nor migration of these cells to extramedullary sites. HSPCs depletion coincided with an increased caspase-3 activation, especially in P-DIE mice. Similarly, the local cytokine milieu in the bone marrow of P-DIE mice was altered to a greater extent compared with P-SUR. Altogether, our study indicates important outcome-related disturbances in hematopoiesis that can inform regarding any future targeted therapies aimed at correcting sepsis-induced myelosuppression.

## Data Availability Statement

The raw data supporting the conclusions of this article will be made available by the authors, without undue reservation.

## Ethics Statement

The animal study was reviewed and approved by Viennese (Austria) legislative committee.

## Author Contributions

TS study design, performing experiments, data analysis/interpretation, and writing manuscript. SD study design, performing experiment, and correction of manuscript. AJ performing experiments. GH study design, performing experiments. MJ sample analysis. JK study design, and writing of the manuscript. MO study design, performing experiments, data analysis/interpretation, and writing manuscript. All authors contributed to the article and approved the submitted version.

## Funding

Study funded by Polish National Science Centre grant no UMO-2016/23/D/NZ6/02554 and Oesterreichs Agentur für Bildung und Internationalisierung (OaeD) grant #PL07/2014.

## Conflict of Interest

The authors declare that the research was conducted in the absence of any commercial or financial relationships that could be construed as a potential conflict of interest.
