# Supplementary material for: An Early Myelosuppression in the Acute Mouse Sepsis Is Partly Outcome-Dependent
Source: Front Immunol. 2021 Jul 22;12:708670. doi: 10.3389/fimmu.2021.708670 (PMC8339578; doi:10.3389/fimmu.2021.708670)
Supplement: Supplementary file 1 [file DataSheet_1.docx]

**ONLINE SUPPLEMENT**

**An early myelosuppression in the acute mouse sepsis is partly outcome-dependent.**

Tomasz Skirecki^1^, Susanne Drechsler^2^, Aldona Jeznach^1^, Grażyna Hoser^1^, Mohammad Jafarmadar^2^, Jerzy Kawiak^1^, Marcin F. Osuchowski^2*^

^1^ Laboratory of Flow Cytometry, Centre of Postgraduate Medical Education, Warsaw, Poland

^2^Ludwig Boltzmann Institute for Experimental and Clinical Traumatology in the AUVA Research Center, Vienna, Austria

Running Title: **Sepsis-induced myelosuppression is outcome dependent**

^*^Corresponding author:

Marcin Osuchowski, PhD, DVM

Ludwig Boltzmann Institute for Experimental and Clinical Traumatology

Donaueschingenstraße 13

A-1200 Vienna, Austria

Tel.: +43 59393

E-mail: marcin.osuchowski@trauma.lbg.ac.at

**Figure Supplemental 1. Outcome-related and longitudinal changes in the circulating cell counts in CD-1 mice subjected to CLP.** Graphs in the upper row (A-D) show comparative data for mice assigned as predicted to survive (P-SUR) and predicted to die (P-DIE). All subgroups were compared using Student t-test with Welch correction whenever required. Graphs in the lower row (E-H) show changes in circulating cells in P-SUR mice only. Groups were compared using 1-way ANOVA with Tukey’s multiple comparison test (for clarity only significance for ANOVA is shown). *p<0.05, **p<0.01, ****p<0.0001

**Figure Supplemental 2**. **Flow cytometry analysis of the bone marrow hematopoietic progenitors.** A. Gating strategy of the multiparametric cytometry is shown. Upper row: mononuclear lineage-negative cells are displayed on ckit vs Sca-1 dot plot to gate common myeloid progenitors (CMP) and LSK cells. Lower row: the LSK cells are further phenotyped using CD48 and CD150 markers to distinguish the long-term hematopoietic stem cells (LT-HSC), short-term HSCs (ST-HSC) and multipotent progenitors (MPP). B. frequencies of the analyzed subpopulations of precursor cells expressed as percentage of mononuclear cells are shown in mice assigned as predicted-to survive (P-SUR) or predicted-to die (P-DIE). Subgroups were compared using Student t-test with Welch correction whenever required. #p<0.05, ##p<0.01. Asterisks indicate a difference versus control (healthy) mice.

**Supplemental Figure 3. Flow cytometry analysis of the blood and spleen hematopoietic progenitors.** Gating strategy of the multiparametric cytometry is presented. A. Peripheral blood analysis. B. Analysis of the splenic cells. In each panel, the upper row shows: mononuclear lineage-negative cells displayed on ckit vs Sca-1 dot plot to gate common myeloid progenitors (CMP) and LSK cells. Lower row: the LSK cells are further phenotyped using CD48 and CD150 markers to distinguish the long-term hematopoietic stem cells (LT-HSC), short-term HSCs (ST-HSC) and multipotent progenitors (MPP)**.**

**Supplemental Figure 4. Flow cytometry analysis of the apoptotic markers in the bone marrow hematopoietic progenitors.** The percentage of LSK cells positive for cleaved caspase-3 as an apoptotic marker.

**Supplemental Figure 5. Longitudinal changes in the cytokine milieu of the bone marrow in P-SUR mice.** Total-protein normalized concentrations of A. IL-6. B. TNF. C. IL-1β. D. IFN-γ. E. IL-5. F. IL-10. G. CXCL1/KC. H. CCL3/MIP-1α. I. CCL2/MCP-1 in the bone marrow supernatants of predicted-to survive (P-SUR) mice subjected to CLP are shown; control (C) n-13, 24h n=10, 48h n=7, 72h n=7, 9d n=5. Concentration of cytokines between groups were compared using ANOVA with Tukey’s post-hoc test. **p<0.01, ****p<0.0001 between control and septic mice. #p<0.05, ##p<0.01, ###p<0.001.

**Supplementary Table 1. Model & Study Design Adherence to MQTiPSS Consensus Recommendations.**

|  | MQTiPSS Recommendations / Considerations | Followed | Explanation |
| --- | --- | --- | --- |
| Study Design | 1. Survival follow-up should reflect the clinical time course of the sepsis model | Y | 1. A nine day follow up |
|  | 1. Therapeutic interventions should be initiated after the septic insult replicating clinical care | Y | 1. Mice given IV fluids and antibiotics (imipenem) 2 hours after infection and administered until day 5 post-CLP |
|  | 1. Treatment should be randomized and blinded when feasible | N/A | 1. N/A – no treatments administered |
|  | 1. Provide as much information as possible (e.g. ARRIVE guidelines) on the model and methodology to enable replication | Y | 1. ARRIVE guidelines followed |
|  | - 1. Consider replication of the findings in models that include comorbidity and/or other biological variables (i.e. age, gender, diabetes, etc.) | N | - 1. Study performed only in female outbred mice without co-morbidities |
|  | - 1. Consider modeling sepsis in other mammals besides rodents | N | - 1. Consideration not met |
|  | - 1. Consider need for source control | N | 1. No source control performed |
| Human Modeling | 1. The development and validation of standardized criteria to monitor the well-being of septic animals | Y | 1. Post-CLP mouse weights, body temperature and body conditioning scores were taken |
|  | 1. The development and validation of standardized euthanasia of septic animals is recommended (exceptions possible) | Y | 1. Mice euthanized based on a custom-developed M-CASS score |
|  | 1. Analgesics recommended for surgical sepsis consistent with ethical considerations | Y | 1. Buprenorphine administered postoperatively and for 5 post-CLP days |
|  | 1. Consider analgesics for nonsurgical sepsis | N | 1. N/A – surgical sepsis model |
| Infection Types | 1. Challenge with LPS is not an appropriate model for replicating human sepsis | Y | 1. Polymicrobial infection from fecal contamination |
|  | 1. Microorganisms used in animal models should preferentially replicate those commonly found in human sepsis | Y | 1. Bacteroides and Firmicutes phyla are the key components of abdominal sepsis in mice and humans |
|  | 1. Consider modeling sepsis syndromes at sites other than the peritoneal cavity | N | 1. Consideration not followed |
| Organ Failure/  Dysfunction | 1. Organ/system dysfunction is defined as life-threatening deviation from normal for that organ/system based on objective evidence | N/A | 1. Organ dysfunction was not an objective of this study |
|  | 1. Not all activities in an individual organ/system need to be abnormal for organ dysfunction to be present | N/A | 1. Organ dysfunction was not an objective of this study |
|  | 1. To define objective evidence of the severity of organ/system dysfunction, a scoring system should be developed, validated, and used or use an existing scoring system | N/A | 1. Organ dysfunction was not an objective of this study |
|  | 1. Not all experiments must measure all parameters of organ dysfunction but animal models should be fully exploited | Y | 1. Animals were exploited with the bone marrow, splenic and blood compartments analyzed |
|  | 1. Avoid hypoglycemia | N | 1. Glucose not monitored |
| Fluid Resuscitation | 1. Fluid resuscitation is essential unless part of the study | Y | 1. Fluids administered 2h post-op and BID thereafter for a total of 5d |
|  | 1. Administer fluid resuscitation based on the specific requirements of the model | Y | 1. Fluid resuscitation tailored to antibiotics requirements (q12hrs) and given postoperatively to offset fluid loss during CLP surgery |
|  | 1. Consider the specific sepsis model for the timing of the start and continuation for fluid resuscitation | Y | 1. Timing of the start and continuation of IVF based on the anticipated clinical course of septic humans |
|  | 1. Resuscitation with iso-osmolar crystalloid solutions is recommended | Y | 1. Normal saline administered (an iso-osmolar crystalloid solution) |
|  | 1. Consider using pre-defined endpoints for fluid resuscitation, as necessary | N | 1. No specific pre-defined endpoints used for fluid resuscitation |
|  | 1. Avoid fluid overload | N | 1. Potential fluid overload not monitored |
|  |  |  |  |
| Antimicrobial Therapy | 1. Antimicrobials are recommended for preclinical studies assessing potential human therapeutics | Y | 1. Antimicrobials administered (imipenem/cilastatin) |
|  | 1. Antimicrobials should be chosen based on the model and likely/known pathogen | Y | 1. Imipenem recommended for intraabdominal sepsis |
|  | 1. Administration of antimicrobials should mimic clinical practice | Y | 1. Antibiotics given BID for 5 days |
|  | 1. Antimicrobials should be initiated after sepsis is established | Y | 1. Imipenem initiated 2h after sepsis onset |

**Supplementary Table 2. Antibodies used in the study.**

| **No** | **Antigen** | **Fluorochrome** | **Clone** | **Working concentration** | **Manufacturer** |
| --- | --- | --- | --- | --- | --- |
| 1 | Lineage Cocktail | APC | 145-2C11 (CD3e), M1/70 (CD11b), RA3-6B2 (CD45R/B220), TER-119 (Ly-76), RB6-8C5 | 20𝜇l/100 𝜇l | BD Pharmingen |
| 2 | c-kit (CD117) | PE | 2B8 | 0.2mg/ml | BD Pharmingen |
| 3 | CD48 | biotin | HM48-1 | 2𝜇g/ml | BioLegend |
| 4 | CD150 | AlexaFluor488 | TC15-12F12.2 | 2𝜇g/ml | BioLegend |
| 5 | Sca-1 (Ly-6A/E) | PE-Cy7 | D7 | 2𝜇g/ml | BD Pharmingen |
| 6 | Active caspase-3 | FITC | C92-605 | 2𝜇g/ml |  |
| 7 | Streptavidin | eFluor610 | - | 1.2 𝜇g/ml | BioLegend |
| 7 | Lineage Cocktail | eFluor405 | CD3 (17A2), CD45R (B220) (RA3-6B2),  CD11b (M1/70), TER-119 (TER-119), Ly-G6 (Gr-1) (RB6-8C5) | 20𝜇l/100 𝜇l | Thermofisher |
